# Dynamics of an estuarine biotic community captured in high spatio-temporal resolution using metabarcoding

**DOI:** 10.1038/s41597-025-06249-1

**Published:** 2025-11-11

**Authors:** T. Sperlea, C. C. Glackin, L. Vogel, E. Zschaubitz, C. Nietz, S. Elferink, C. Loose, H. Schröder, C. Hassenrück, M. Labrenz

**Affiliations:** 1 Leibniz Institute for Baltic Sea Research Warnemünde (IOW), Rostock, 18119 Germany; 2 LGC Genomics GmbH, Berlin, Germany; 3 Planet AI GmbH, Rostock, Germany

**Keywords:** Biodiversity, Molecular ecology

## Abstract

Estuaries and coasts are dynamic transition zones linking freshwater and marine environments and are characterized by sharp physicochemical gradients. These regions support key ecosystem functions but are challenging to study due to their spatial and temporal variability. Here we present a dataset characterizing microbial community composition and environmental parameters across the Warnow River estuary and adjacent Baltic Sea coast in unprecedented spatio-temporal resolution. Over the course of a year, we sampled fifteen sites along a ~30 km transect up to twice per week, generating 16S and 18S rRNA amplicon sequencing data, flow cytometry profiles, and measurements of temperature, salinity, chlorophyll a, nitrate, nitrite, ammonium and phosphate concentrations. Spanning the salinity gradient from freshwater to brackish environments, this dataset resolves short-term dynamics and fine-scale spatial variation in microbial communities within a human-impacted temperate estuary. It enables the investigation of microbial dynamics, community turnover, and biogeochemical responses to environmental change, offering a valuable resource for modeling microbial processes in complex, human-impacted aquatic systems.

## Background & Summary

Many major cities worldwide are located in estuaries and coastal regions^1^. The development of these settlements is historically entangled with the ecosystem services provided by estuaries and coasts, including fishing, commerce and trade, cargo transport, tourism and recreation^2^. At the same time, estuarine and coastal cities can exert significant pressure on the ecosystems surrounding them^3^. Courses and beds of estuaries are often deepened and fortified to better fit the requirements of ship transport, with a considerable effect on water currents and benthic communities^4,5^. Waste water treatment plants introduce pharmaceuticals, nutrients, and other anthropogenic trace substances from the city into the river^6^. Cargo transport is a vector for the transmission of invasive species^7^ and tourism can lead to elevated levels of pollution by waste and noise, and the destruction of local wildlife^8,9^. The interdependence between the anthropogenic and environmental factors highlights that the estuary and the coastal cities located within them are coupled social-ecological systems, in which the dynamics of one domain cannot be fully understood in isolation from the other^10,11^.

In addition to seasonal variation and weather events, the dynamics of biotic communities in estuaries are shaped by the fact that they inhabit transition waters connected to both the riverine and marine environment^12–14^. The resulting salinity gradient is highly dynamic and subject to transitional mixing that is driven by the interplay between the water density gradient, wind, and tidal dynamics and can, therefore, change directions multiple times a day at small scales^15^. The hydrological inhomogeneity of estuarine waters imposes a constant natural stress on the biotic community and complicates the detection and attribution of anthropogenic stressors^16^ It is thus not surprising that salinity and seasonality are known to be major factors shaping the biotic community of both brackish as well as estuarine ecosystems^17–21^. In addition, many estuarine systems host urban and industrial hubs that required geomorphological modifications to channels and shorelines, often resulting in substantial shifts in biodiversity relative to historical baselines^1^.

With around 21.5 megatonnes of cargo processed annually in Rostocks harbors and 2.7 million passengers utilizing its waterways to or from the 210.000 inhabitants city Rostock, the Warnow estuary has a high socio-economical value for the whole northeastern region of Germany^22^. It provides a wide array of ecosystem services, including navigation, maritime industry, energy production, nursery grounds and habitats for fish, aesthetic experiences and a wide range of leisure activities^23^. The water in the estuary is highly dynamic with surface mixing between fresh water from the Warnow river and brackish water from the Baltic Sea being controlled by wind conditions, limited tidal influence, and a flushing time of approximately 30 days^15^. As the estuary bottom is, with a maximum depth of 15 m, below the Baltic Sea level, a considerable amount of brackish water flows into the estuary in dry periods^12^. The surrounding coastal zone near Rostock encompasses diverse geomorphological features, including densely visited sandy beaches, forested cliffs bordering a protected area, and a historically modified estuarine system adapted for both inland and maritime navigation since the 13th century^24,25^. Multiple sources of anthropogenic stress impact the region, imported from wastewater treatment discharge, the Baltic Sea and from the Warnow river catchment. Notably, previous investigations have assessed the concentrations of pharmaceuticals and personal care products^26^ as well as microplastic contamination^27^ within the Warnow estuary and the adjacent coastal zone.

**Fig. 1:**
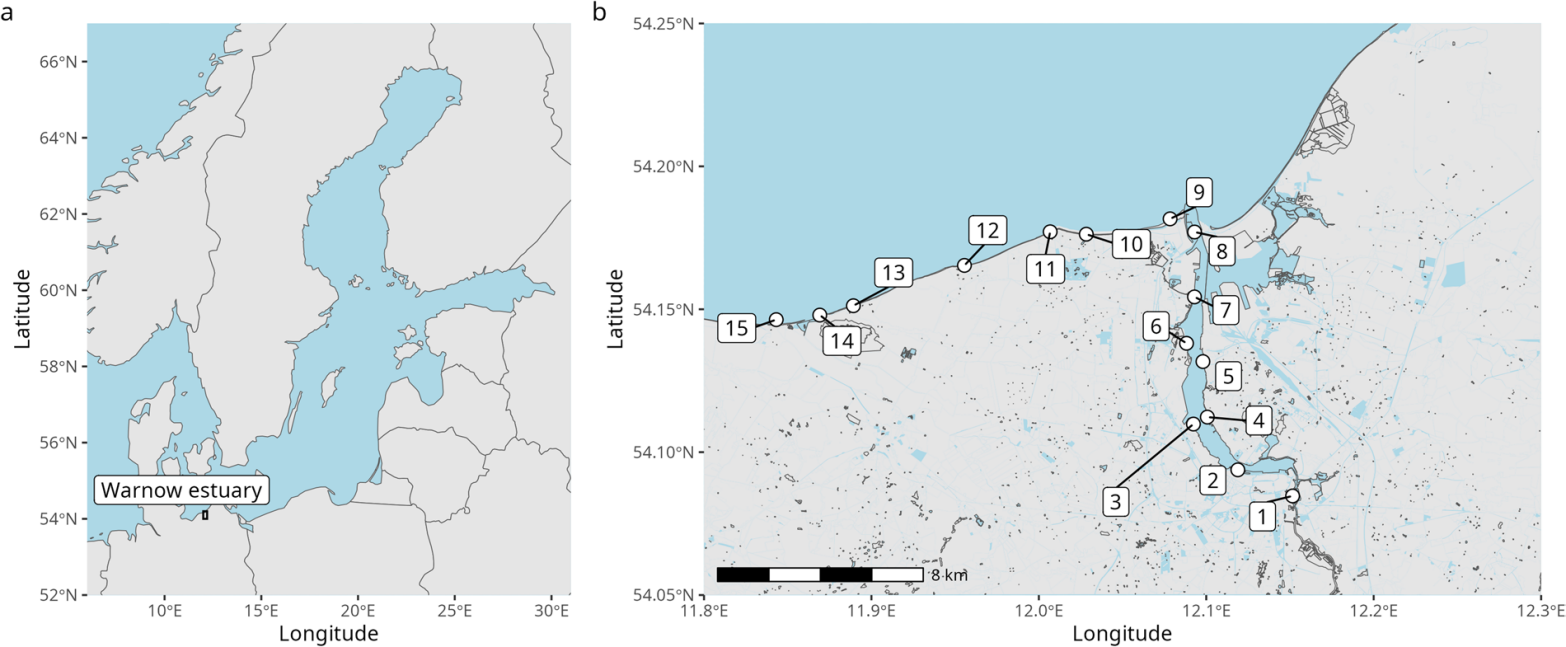
Geographic context (**a**) and location of the sites sampled in this study (**b**).

Here we present a dataset describing the dynamics in the biotic community of the estuarine and brackish coastal environment closely coupled to the city of Rostock. We analyzed surface water samples taken twice a week at fourteen sites and weekly at one additional site along the salinity gradient from fresh water from the Warnow River to the brackish water of the Baltic Sea (Fig. 1), leading to a data set with an unprecedented temporal and spatial resolution. All samples are surface water samples taken “from land” using a metal bucket and at times fixed relative to the sunrise to control for diurnal effects (see Methods, subsection “Sampling”). The data presented here and the concentrations of anthropogenic trace substances recorded and discussed in ref. ^28^ come from the same samples. Furthermore, one sampling site included in this dataset is the sampling site of the Heiligendamm time series, which records the presence and number of phytoplankton at the Baltic Sea coast in a weekly rhythm since 1988 using microscopic methods^29,30^, enabling contextualization of this dataset within long-term ecological records.

**Fig. 2:**
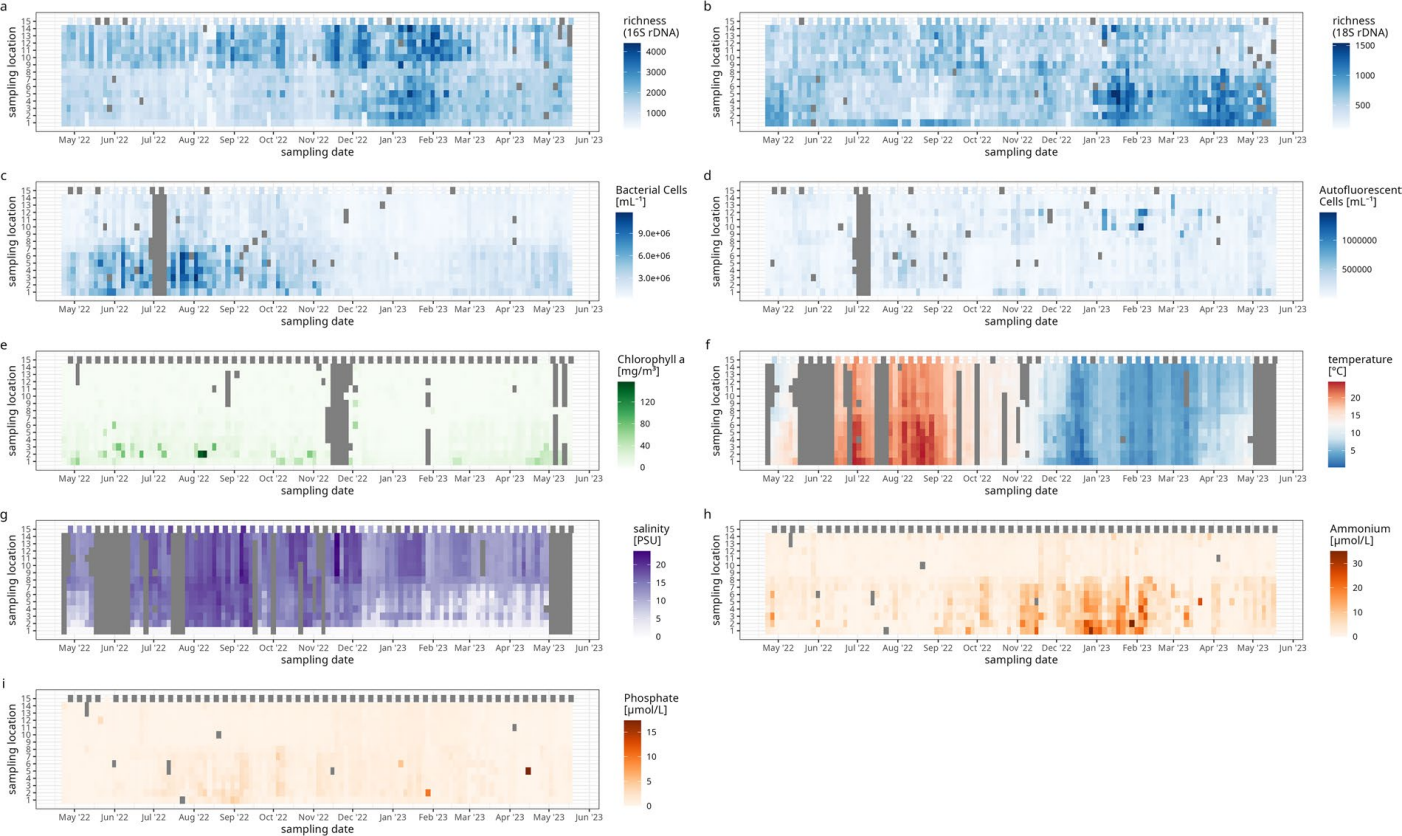
Spatio-temporal dynamics of species richness as determined using 16S (**a**) and 18S (**b**) rRNA gene metabarcoding, cell counts of heterotrophic (**c**) and autofluorescent cells (**d**), chlorophyll a concentrations (**e**), temperature (**f**), salinity (**g**), ammonium (**h**) and phosphate concentrations (**i**).

**Fig. 3:**
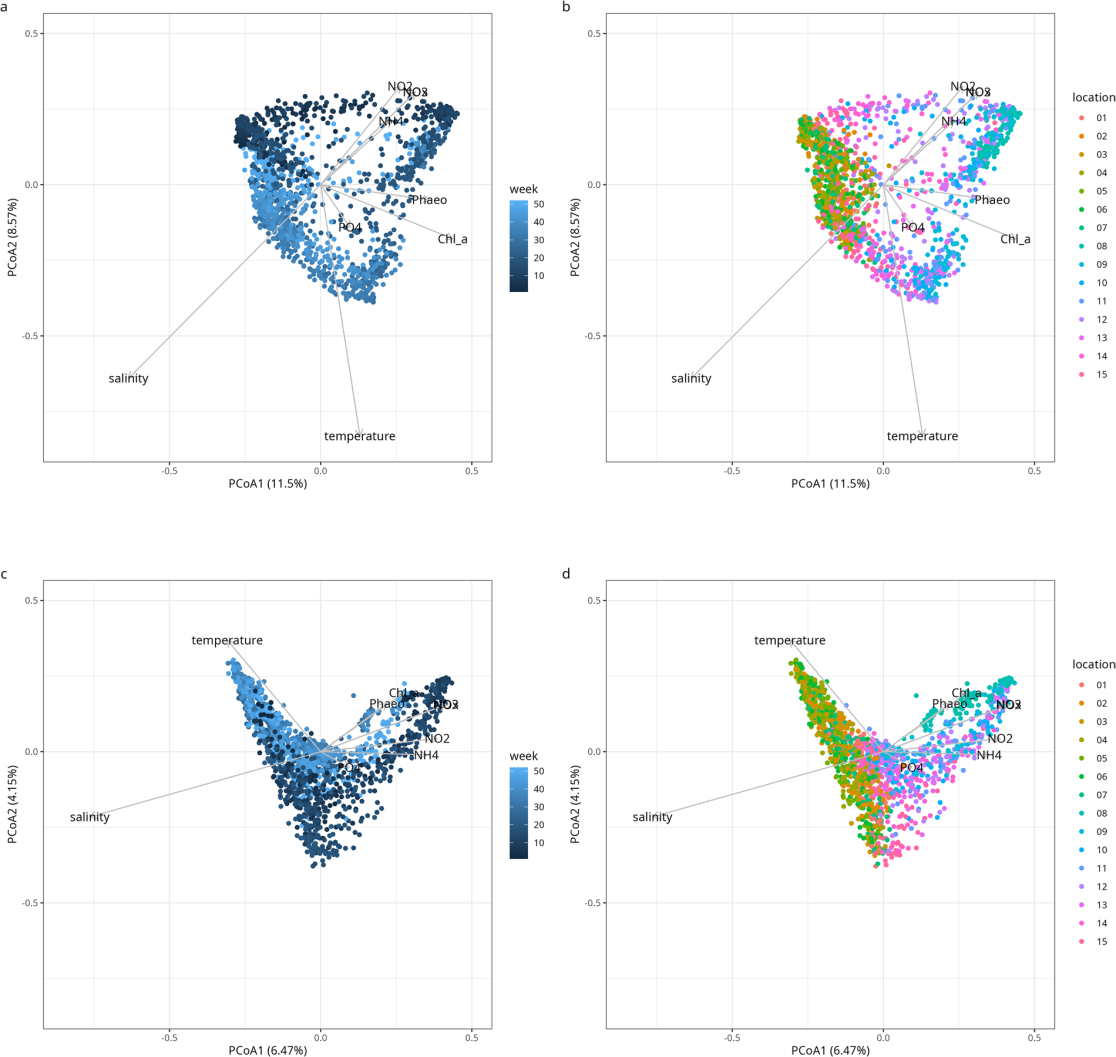
PCoA ordination based on the Bray-Curtis dissimilarities for 16S (**a,b**) and 18S (**c,d**) rRNA gene metabarcoding shows temporal (**a,c**) and spatial patterns (**b,d**). Points represent samples and arrows represent abiotic and biotic factors.

**Fig. 4:**
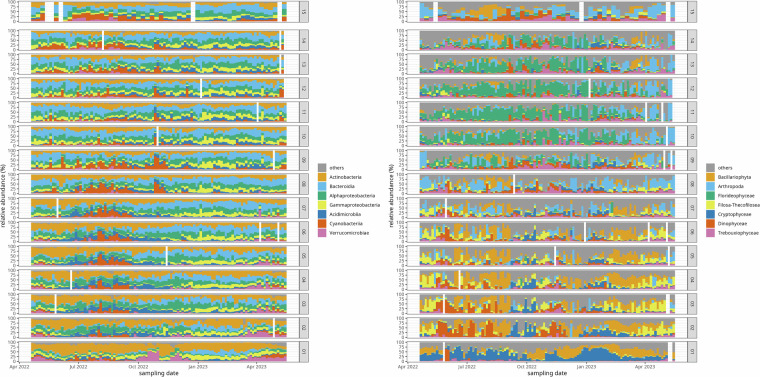
Taxonomic composition of the Warnow estuary and Baltic Sea coast biotic community. Shown are the 7 classes with the highest relative abundance over all samples for 16S (**a**) and 18S (**b**) rRNA gene metabarcoding.

To capture the composition of the biotic community in a taxonomic range as wide of as possible, we performed metabarcoding (also known as amplicon sequencing) with broad-range primer sets targeting the 16S and 18S rRNA genes for prokaryotes and eukaryotes, respectively (Fig. 2a,b, see Methods, section “DNA extraction and sequencing” for details). To capture total and group specific cell counts for phototroph cells in a high-throughput setting, we performed Flow Cytometry (Fig. 2c,d). Flow Cytometry data can be used to quantify the abundance of taxa assessed via amplicon sequencing in absolute terms^31^. To assess the abiotic context of the samples, we measured salinity, temperature, chlorophyll a, nitrate, nitrite, ammonium and phosphate concentrations (Fig. 2e–i). In all variables captured in this data set, we observe spatial and seasonal variation (Figs. 2, 3). For example, the community composition mirrors the salinity gradient as amplicons from Actinobacteria, Verrucomicrobiae, Bacillariophyta, Filosa-Thecofilosea, and Cryptophyceae dominate the fresh water samples whereas amplicons from Bacteroidia, Cyanobacteria, Florideophyceae and Arthropoda (mainly from the genera *Pseudocalanus*, *Centropages*, and *Acartia*) dominate the Baltic Sea samples at different points of the annual cycle (Fig. 4).

## Methods

### Sampling area

The Warnow river is 155 km long and drains a catchment of around 3.300 km² that is affected by agriculture and, thus, highly eutrophied^20^. A weir actively controls the inflow of freshwater into the estuary and isolates the freshwater river from the reflux of more saline water from the estuary. Sampling site 1 of this dataset is located just upstream of this weir, in an area surrounded by gardens and adjacent to a small public swimming site that is open only in summer (Fig. 1). Downstream of the weir, the Warnow sharply turns westward and widens into a basin with a maximum width of about 540 meters. Sampling site 2 is located at the tip of a pier for smaller ships in the city harbor in this basin. Sampling site 3 is located downstream of the discharge point of Rostock’s waste water treatment plan and site 4 is located at the opposing riverbank of the Warnow estuary and may act as a control site. Roughly 3 km further downstream, sampling site 5 is positioned at a pier adjacent to a boat refueling station on the eastern bank of the Warnow. On the western riverbank, sampling site 6 is located at a pier for smaller vessels near a small sandy beach and a water sports facility popular in the summer. Sampling site 7 is located at the inflow of the small river Laak into the estuary, opposite Rostocks large oversea harbor. Sampling site 8, located at the 1.15 km wide mouth of the Warnow estuary, is expected to reflect the strongest influence of ship traffic and cargo transport, being positioned just downstream of the anchorage area for cruise ships and the ferry terminal serving Hohe Düne.

Whereas estuarine samples were taken by lowering a metal bucket into the water, coastal samples were taken by wading into the water to a depth of ~1 m. Sampling site 9 is located at the main sandy beach of Warnemünde, a highly frequented bathing area during the summer months that attracts both tourists and local residents. Given a high river discharge and easterly winds, this sampling station can be affected by the Warnow river plume. Progressing westward along the coast, sampling sites 10 (near a small hotel and restaurant, after 3.5 km) and 11 (after 5.4 km) are located at stonier and less popular beaches and adjacent to a small forest. Sampling sites 11 and 12 are positioned next to cliffs that are stabilized and covered by trees; the latter, being less accessible, experiences less human activity than the former. Sampling site 13 is located at a beach popular for bathing and small boats close to the small municipality Börgerende-Rethwisch. At site 14, samples are taken on the western side of the large stone structure erected at the mouth of the small river Mühlenfließ, which serves as the deferent of treated waste water from the city of Bad Doberan. Finally, sampling site 15 is positioned at the tip of the Heiligendamm pier and corresponds to the location of the long-term Heiligendamm phytoplankton time series.

### Sampling

Surface water sampling was performed using a metal bucket lowered into the sea using a rope (sampling sites 8 and 15), by hand (sites 1–7), or while standing in the water (sites 9–14). Sampling began each day at site 14, approximately three hours after sunrise, proceeded westward along the coast to site 9, and then continued sequentially from site 1 through site 8. Samples were transported on ice and up to 500 mL were filtered in the lab using a Durapore PVDF filter with a pore size of 0.22 μm and a diameter of 47 mm. Filters were cut in half and separately frozen in liquid nitrogen and kept at −80 *C until further processing. Genomic DNA was extracted from the filters using the KingFisher Flex purification system and the MagMAX Wastewater Kit after bead beating with MagMAX Microbiome silica beads and 800 μL lysis buffer at 2500 rpm for 10 min.

### DNA extraction and sequencing

Paired-end DNA amplicon sequencing was performed by LGC Genomics (Berlin, Germany) on an Illumina MiSeq. For the metabarcoding of the prokaryotic community the V3-V4 hypervariable region of the 16S rRNA gene was sequenced using the primers 5’-CCTACGGGNGGCWGCAG-3’ and 5’-GACTACHVGGGTATCTAAKCC-3’^18^; the eukaryotic community was assessed based on the V4 hypervariable region of the 18S rRNA gene using the primers 5’-CCAGCASCYGCGGTAATTCC-3’ and 5’-ACTTTCGTTCTTGATYRR-3’^32^. These primers were chosen because of their high taxonomic coverage (see Technical Validation). Demultiplexed and adapter-clipped sequence reads provided by LGC were further processed as documented here: https://git.io-warnemuende.de/bio_inf/IOWseq.000042_OTCgenomics_seqs. Briefly, primers were removed with cudadapt version 4.2^33^ allowing a mismatch rate of 0.16. Reads that did not match the primer sequence and reads shorter than 50 bp were discarded. The following analysis steps were conducted in R version 4.2.2^34^ using the dada2 package version 1.26.0^35^. R1 and R2 reads were truncated to 260 bp and 200 bp, respectively, and quality filtered at a maximum expected error rate of 3 bp. Error learning and denoising were performed in batches of 96 samples that were sequenced at the same time. The 16S data set was generated from mixed orientation libraries (where the R1 read file contained both forward and reverse reads, and vice versa) each orientation was processed independently. For the 18S data set, a modified version of the error learning function was applied to avoid underestimation of error rates in the error model. All sequences per batch were pooled for denoising to generate amplicon sequence variants (ASVs). Merging of paired-end reads was performed with a minimal overlap of 10 bp and zero mismatches. Prior to chimera removal with default settings, reverse-forward oriented 16S ASVs were reverse-complemented and combined with the ASV count table generated from the forward-reverse oriented reads of each batch. Finally, ASVs were taxonomically classified using the RDP Bayesian classifer implemented in dada2 with the SILVA Ribosomal Reference Database version 138.1^36^ and the Protist Ribosomal Reference Database (PR2) version 4.14.0^37^ for 16S and 18S ASVs, respectively. ASVs unclassified at domain level as well as those classified as chloroplast and mitochondria in the 16S data set were discarded from the further analysis. For each marker gene, batches were combined into a common ASV count table containing the community profiles of all samples after the completion of the bioinformatic processing pipeline.

### Processing of sequencing data

Alpha diversity metrics were calculated using the iNEXT function from the iNEXT package (version 3.0.1)^38,39^ with the Hill numbers 0 and 2, an endpoint of 20.000 and 20 knots. For all betadiversity analyses, we applied a rarity filter, excluding ASVs that were not present in at least 1% of samples with 0.1% of relative abundance. Bray Curtis distance matrices and PCoA ordinations were calculated using the functions vegdist, envfit and betadisper from the package vegan (version 2.6.6)^40^.

### Measurement of physical and chemical parameters and chlorophyll

Temperature and salinity were measured in the field using either a CTD75M or a CTD48M (Sea and Sun Technology, Germany) probe directly in the bucket. Chlorophyll a concentrations were measured using 500 mL of the sample that were filtered using a Ø47 mm GF/F filter and a 0-AU-005-CE fluorometer (Turner) following the method described in ref. ^41^. Ammonia, phosphate, nitrite and nitrate concentrations were measured using the continuous segmented flow analyzer QuAAtro 39 (SEAL Analytical) following ref. ^42^.

### Flow cytometry

Bacterial and autofluorescent cells were counted following ref. ^43^. After transporting the samples to the laboratory, for each sample, 2 × 4 mL of the sample were mixed with 200 μL 30% formol in a cryovial, incubated at 4 °C in the dark for 1 h, shock frozen in nitrogen and stored at −80 °C until further analysis. Samples for autofluorescent cells were counted without further staining and, if necessary, diluted in relation to desired concentration for comparable measurements. Samples for heterotrophic cells were stained with 22 μL SYBR Green I (Invitrogen, Waltham, USA) and diluted with MQ water with a 1:10 Dilution factor. All measurements were performed using a CytoFLEX S flow cytometer (Beckman coulter, Brea, USA) at 30 μL/mL flow rate for 3 minutes and using 10 μL yellow latex beads (0.88 μm, Spherotech) in each sample as internal standard. The software CytExpert (Version 2.6.0.105, Beckman Coulter Inc.) was used for gating of the samples.

## Data Record

Demulitplexed and primer-clipped read data are available from the European Nucleotide Archive (ENA) with MIxS compatible metadata^44^ using the data brokerage service GFBio^45^ and are available under the study accession numbers PRJEB88008^46^ (16S) and PRJEB88011^47^ (18S) (Table 1). Flow cytometry and contextual data are publicly stored in the IOWDB and can be accessed via the DOIs 10.12754/data-2025-0005^48^ and 10.12754/data-2025-0001^49^. Derived and clean datasets including ASV tables are available at figshare^50^. Common sample IDs are used across all repositories to facilitate sample re-identification.

**Table 1:** Overview over the data records.

Data type	Structure	Modalities/Variables	Repository
16 s rRNA amplicons	Two runs (mixed orientation) per sample plus mock communities and blanks	Raw sequencing data	ENA, PRJEB88008^46^
18 s rRNA amplicons	One run per sample plus mock communities and blanks	Raw sequencing data	ENA, PRJEB88011^47^
Flow cytometry data	Two files (autofluorescent and heterotrophic cells) per sample	Raw flow cytometry data	IOWDB, 10.12754/data-2025-0005^48^
Contextual data	One tabular file per location, a line per sample	location_id, lat, lon, unix timestamp, sampling date, time, phosphate (PHOS7DXD, mmol/m^3^), ammonium (AMON7DXD, mmol/m^3^), nitrite (NTRI7DXD, mmol/m^3^), nitrate (NTRA7DXD, mmol/m^3^), chlorophyll a (CPHT2F2D, mg/m^3^) concentrations, temperature (TEMP7IDD, °C), salinity (PSAL7IDD)	IOWDB, 10.12754/data-2025-0001^49^
Code and derived data	One table per data modality, one line per sample	Autofluorescent and heterotrophic cell counts, full and rarity filtered OTUs and taxonomic annotation of OTUs derived from the amplicon data	figshare^50^

## Technical Validation

**Fig. 5:**
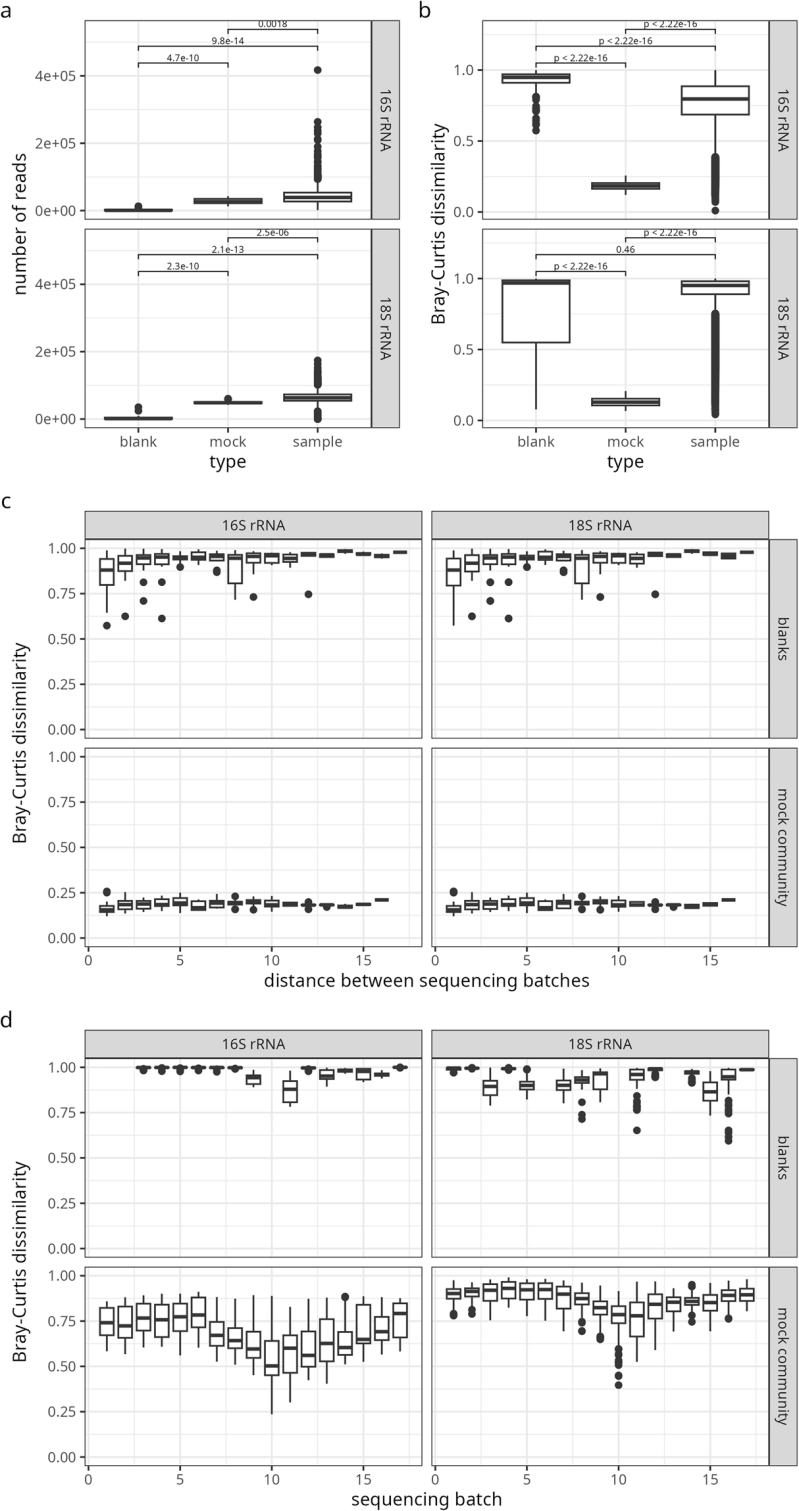
Validation of sequencing runs via blank and mock community samples. (**a**) Number of reads in and (**b**) Bray-Curtis dissimilarity among blank, mock community and real samples. (**c**) Bray-Curtis dissimilarity between blank and mock community samples between batches and (**d**) between blank or mock community samples and environmental samples in the same sequencing batch.

To validate the metabarcode sequencing process, we added a blank (sterile water) and a mock community sample to each sequencing run. Blank samples consisted of DEPC-treated water and sequencing them resulted in a significantly lower number of reads than either the samples or the mock community runs (Fig. 5a) and a high level of heterogeneity among blank runs (Fig. 5b). When compared to the samples sequenced in the same run as the blank, we observe a Bray-Curtis dissimilarity of >0.9 for all but a few samples (Fig. 5c) and we do not observe a trend in Bray-Curtis dissimilarity in blanks across batches (Fig. 5d). The mock community was generated by combining subsamples from all samples taken November 7^th^, 2022, across the sampling area, to capture a broad but realistic and comparable community composition. Aliquots of the same mock community DNA extract were used for each sequencing run. Sequenced mock samples show a number of reads comparable to the samples (Fig. 5a), a consistently low Bray-Curtis dissimilarity among mock community runs (Fig. 5b,c) and a reasonably high dissimilarity (25th and 75th percentiles of the Bray-Curtis distance distribution at 0.6 and 0.81 and 0.83 and 0.92 for 16S and 18S rRNA data, respectively) to the samples in each batch that is modulated by seasonal effects (Fig. 5d). Taken together, these results validate the sequencing runs. The consistency of the samples with regard to spatial and temporal patterns was validated manually on the basis of PCoA and NMDS plots based on Bray Curtis dissimilarity matrices and species richness. A total of 18 samples with inconsistent community compositions was removed from the data set.

Flow Cytometry data generation was validated for consistency by using latex beads as internal standard (see Methods for details). Ammonia, phosphate, nitrite and nitrate concentrations were validated through negative and positive controls of known concentrations in all measurement runs. Chlorophyll a concentrations were measured using a method well-established at the Leibniz-Institute for Baltic Sea Research, the validity of which is regularly tested via inter-laboratory controls.

**Fig. 6:**
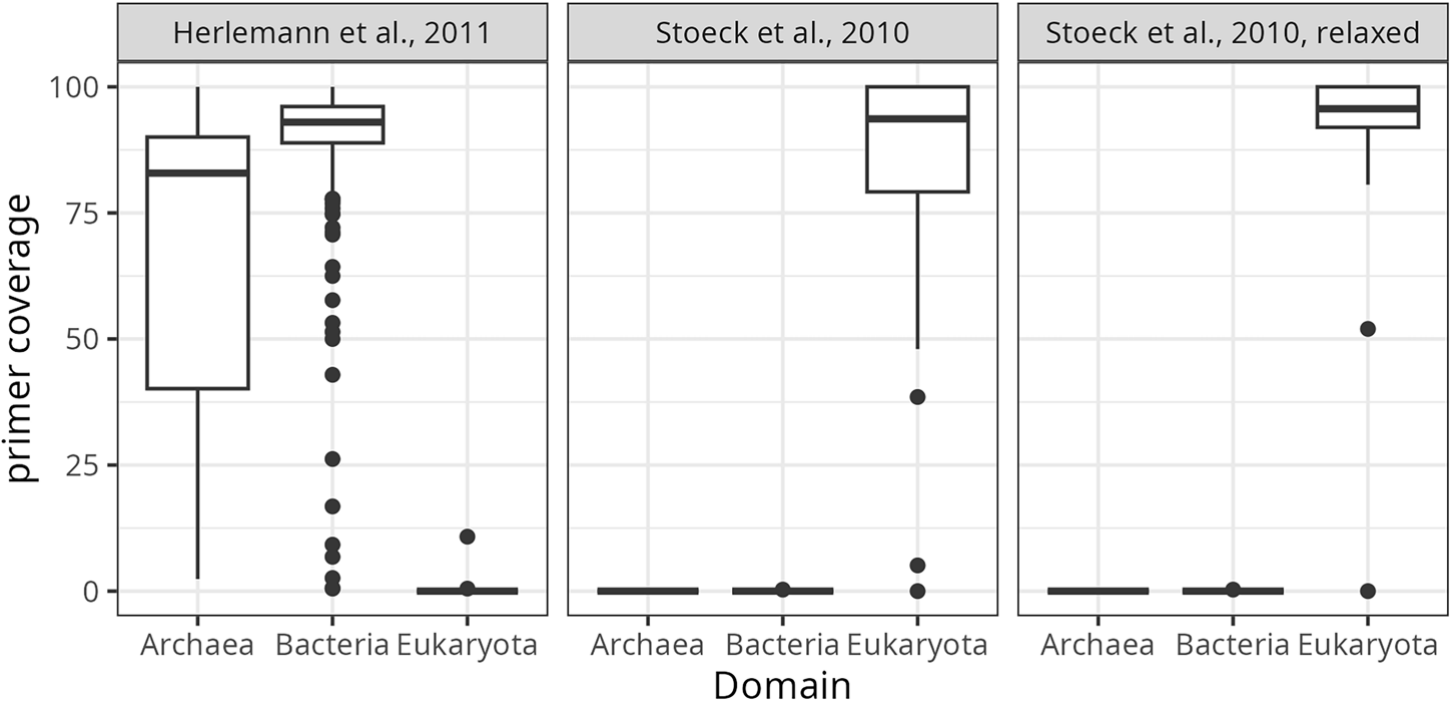
Primer coverage of primers used in this study as identified via *in silico* PCR. Each dot represents a phylum of the respective domain.

The taxonomic coverage of primers used here was evaluated using SILVA TestPrime (date accessed 28.04.2022)^51^ against the non-redundant version of the SILVA ribosomal reference database version 138.1 (SSU RefNR)^36^, allowing either 1 or 3 mismatch(es) with a 5 bp or 3pb zero mismatch zone at the 3’ end of the primer for a stringent and relaxed primer coverage assessment, respectively (Fig. 6).

## Data Availability

16S rRNA gene reads are available under the study accession number PRJEB88008^46^; 18S rRNA gene reads are available under the study accession number PRJEB88011^47^. Flow cytometry and contextual data can be accessed via the DOIs 10.12754/data-2025-0005^48^ and 10.12754/data-2025-0001^49^.

## Code availability

Code used for sequence procession is available at https://git.io-warnemuende.de/bio_inf/IOWseq.000042_OTCgenomics_seqs/releases/download/datapub-1.0.0/datapub-1.0.0.zip. Code used to generate the figures in this manuscript is available at figshare^50^.
